# High-throughput clone library analysis of the mucosa-associated microbiota reveals dysbiosis and differences between inflamed and non-inflamed regions of the intestine in inflammatory bowel disease

**DOI:** 10.1186/1471-2180-11-7

**Published:** 2011-01-10

**Authors:** Alan W Walker, Jeremy D Sanderson, Carol Churcher, Gareth C Parkes, Barry N Hudspith, Neil Rayment, Jonathan Brostoff, Julian Parkhill, Gordon Dougan, Liljana Petrovska

**Affiliations:** 1 Wellcome Trust Sanger Institute, Wellcome Trust Genome Campus, Hinxton, Cambridge, CB10 1SA, UK; 2 King's College London, Biomedical & Health Sciences, Dept. of Nutrition and Dietetics, Franklin-Wilkins Building, 4th floor, 150 Stamford Street, London, SE1 8NH, UK; 3 Department of Bacteriology, Veterinary Laboratories Agency (Weybridge), Woodham Lane, Addlestone, Surrey, KT15 3NB, UK

## Abstract

**Background:**

The gut microbiota is thought to play a key role in the development of the inflammatory bowel diseases Crohn's disease (CD) and ulcerative colitis (UC). Shifts in the composition of resident bacteria have been postulated to drive the chronic inflammation seen in both diseases (the "dysbiosis" hypothesis). We therefore specifically sought to compare the mucosa-associated microbiota from both inflamed and non-inflamed sites of the colon in CD and UC patients to that from non-IBD controls and to detect disease-specific profiles.

**Results:**

Paired mucosal biopsies of inflamed and non-inflamed intestinal tissue from 6 CD (n = 12) and 6 UC (n = 12) patients were compared to biopsies from 5 healthy controls (n = 5) by in-depth sequencing of over 10,000 near full-length bacterial 16S rRNA genes. The results indicate that mucosal microbial diversity is reduced in IBD, particularly in CD, and that the species composition is disturbed. *Firmicutes *were reduced in IBD samples and there were concurrent increases in *Bacteroidetes*, and in CD only, *Enterobacteriaceae*. There were also significant differences in microbial community structure between inflamed and non-inflamed mucosal sites. However, these differences varied greatly between individuals, meaning there was no obvious bacterial signature that was positively associated with the inflamed gut.

**Conclusions:**

These results may support the hypothesis that the overall dysbiosis observed in inflammatory bowel disease patients relative to non-IBD controls might to some extent be a result of the disturbed gut environment rather than the direct cause of disease. Nonetheless, the observed shifts in microbiota composition may be important factors in disease maintenance and severity.

## Background

Inflammatory bowel disease (IBD) encompasses both Crohn's disease (CD) and ulcerative colitis (UC), chronic inflammatory disorders of the gastrointestinal tract with developed world predominance and an incidence that has risen dramatically in the post-war period [1]. IBD manifests with symptoms such as severe diarrhoea, weight loss and debilitating abdominal pain, resulting in substantial morbidity and impairment in quality of life [2]. In both diseases visibly inflamed and non-inflamed areas of intestine can be identified at assessment by colonoscopy.

The cause of both conditions is still speculative. Host genetics play a key role, with genetic factors more important for development of CD than UC [3,4], but genetic defects cannot wholly explain the increasing prevalence of IBD in recent years, suggesting that environmental factors are also involved [5]. The current generally accepted disease hypothesis is that the chronic inflammation of IBD results from a genetically dysregulated host immune response directed at the gut microbiota [6-8].

The human gut microbiota is a highly diverse and abundant community of microbes that under normal circumstances is either commensal or beneficial to human health [9]. Bacteria in the gut contribute to host nutrition via production of short chain fatty acids and vitamins, and play integral roles in maintaining human health by preventing colonisation by pathogens and by shaping and maintaining normal mucosal immunity [10]. The microbiota is also, however, a major source of antigens, including lipopolysaccharides, peptidoglycan, lipoproteins, flagellin and unmethylated CpG-containing DNA, all of which can activate both innate and adaptive immune responses [11,12]. A balanced relationship, therefore, must exist between bacteria and their human hosts. A disruption in this homeostasis threatens the state of immune tolerance and may result in gut inflammation.

Several lines of evidence suggest a role for gut bacteria in the pathogenesis of IBD. Faecal stream diversion induces remission in CD [13], animal models of colitis require the presence of gut bacteria to initiate inflammation (reviewed in [14]), an increased mucosal bacterial load is observed in IBD patients [15,16], genome-wide IBD association studies have identified polymorphisms in genes involved in bacterial recognition and clearing (reviewed in [17]) and broad-spectrum antibiotics have some efficacy in the treatment of CD [18,19].

With CD in particular, individual species such as *Mycobacterium avium *subspecies *paratuberculosis *or *Escherichia coli *have been implicated in disease aetiology [20,21] while the emerging "dysbiosis" hypothesis implicates multi-species assemblages in an overall imbalance between harmful and protective bacteria [22,23]. Numerous studies have attempted to characterise the microbial communities in IBD and to compare these with healthy individuals. Results indicate that individuals with IBD have reduced bacterial diversity, temporal stability and cluster separately when compared to healthy controls [24-28]. Compositional comparisons have generated inconsistent results but have generally identified reductions in components of the *Firmicutes *phylum in IBD, often, but not always, with concurrent increases in *Bacteroidetes *and facultative anaerobes such as *Enterobacteriaceae *[12,22,29-31].

Faecal/luminal bacterial communities have repeatedly been shown to be distinct from mucosal communities [32-37], meaning that study of the IBD mucosa-associated microbiota and comparison with those from healthy individuals should provide the best insight into whether or not a particular microbial signature is disease specific. In addition, within IBD-affected intestines disease-causing agents might be enriched at sites of active inflammation relative to comparatively unaffected mucosa. We have therefore used in-depth bacterial 16S rRNA gene cloning and sequencing technology to compare the mucosa-associated microbiota from inflamed and non-inflamed sites of the colon in CD and UC patients and in non-IBD controls. Our findings indicate that mucosal microbial diversity and composition is disturbed in IBD and that there are significant differences in microbial community structure between inflamed and non-inflamed mucosa.

## Results

Twenty-nine mucosal biopsies were collected from a total of seventeen patients, including paired biopsies of inflamed and non-inflamed tissue from six patients with active CD (n = 12), paired biopsies from six patients with active UC (n = 12) and five biopsies from non-IBD controls (n = 5). Demographic data, disease phenotype, biopsy site and histological scores are shown in Table 1. All biopsies from non-IBD controls were histologically normal. There was no age difference between CD and UC cases but, due to the indication for colonoscopy, the average age of the non-IBD control patients was higher. The median ages were 32 (25-51) years for the CD group, 26 (24-73) years for the UC group and 51 (45-73) years for the controls. Disease duration was similar.

**Table 1:** Characteristics of patients and biopsy tissue at time of sampling.

Diagnosis	**No**.	Age	Sex	Biopsy Site	Baron Score	Biopsy site	Baron Score
CD	1	51	M	Rectum	3	Descending	0
CD	2	25	F	Descending	2	Descending	0
CD	3	35	F	Sigmoid	3	Descending	1
CD	4	29	F	Transverse	2	Sigmoid	0
CD	5	35	F	Sigmoid	2	Transverse	0
CD	6	26	M	Transverse	3	Sigmoid	0
UC	1	49	M	Sigmoid	1	Transverse	0
UC	2	26	M	Sigmoid	2	Sigmoid	0
UC	3	73	M	Rectum	1	Descending	0
UC	4	25	M	Transverse	2	Ascending	0
UC	5	26	M	Sigmoid	2	Splenic	0
UC	6	24	F	Rectum	2	Descending	0
Non-IBD	1	72	F	n/a	n/a	Sigmoid	n/a
Non-IBD	2	51	F	n/a	n/a	Rectum	n/a
Non-IBD	3	48	F	n/a	n/a	Rectum	n/a
Non-IBD	4	45	M	n/a	n/a	Terminal Ileum	n/a
Non-IBD	5	73	M	n/a	n/a	Descending	n/a

### Quantification of bacterial populations

**Figure 1:**
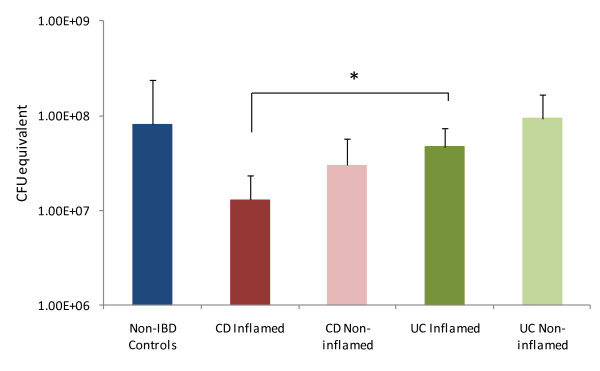
**qPCR analysis of total bacterial load in mucosal biopsy samples**. Figures are mean results for each patient cohort. Error bars denote standard deviation from the mean. Total bacterial load was significantly lower in the inflamed CD biopsies than the UC inflamed biopsies.

Using qPCR we measured the total bacterial load in the mucosal biopsy samples. The results showed high variability between samples but overall the biopsies from the inflamed intestinal regions of CD patients contained the lowest number of bacteria (Figure 1). The total number of bacteria detected in these inflamed CD samples was significantly lower than the bacterial load present in the inflamed regions of the UC patients' colons. While it appeared that within each disease cohort the bacterial load was generally lower in inflamed regions of the colon compared to non-inflamed regions the inter-individual variation meant that no other significant differences were detected.

### Overall phylogenetic classification of 16S rRNA gene sequences

We next analysed the bacterial diversity in the 29 mucosal biopsy samples by deep sequencing of 16S rRNA gene clone libraries. The final dataset of 10,010 chimera-checked, full-length sequences included an average of 620 clones per CD patient, 750 clones per UC patient and ~350 clones per healthy control. As a whole, the dataset contained an estimated 565 phylotypes (clustered at >99% sequence identity), which could be mapped to eight bacterial phyla. 93% of the sequences belonged to just two of these phyla; the *Firmicutes *(51.8% of clones) and the *Bacteroidetes *(41.1%). Within the *Firmicutes *phylum the vast majority of sequences grouped into two families, the *Lachnospiraceae *(51.2%) and the *Ruminococcaceae *(33.1%), which comprise clostridial clusters XIVa and IV respectively. The *Bacteroidetes *sequences were predominantly from the *Bacteroidaceae *family (62.6%) but also included *Porphyromonadaceae*, mainly *Parabacteroides *species, (13%) and *Prevotellaceae *(19%). *Proteobacteria *represented ~6% of the total sequences, the majority of which were β-proteobacterial species related to *Sutterella *spp. The remaining five phyla we detected each accounted for less than 1% of total bacteria: *Actinobacteria *(0.89%), *Fusobacteria *(0.14%), *Verrucomicrobia *(0.03%), *Lentisphaera *(0.01%) and TM7 bacteria (0.02%).

### Comparison of bacterial composition in IBD and control biopsies

**Figure 2:**
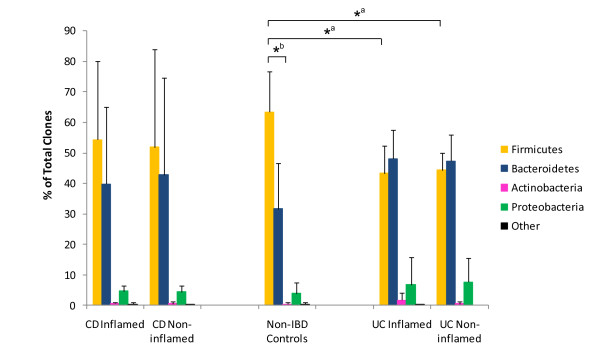
**Compositional analysis of 16S rRNA gene clone libraries**. Phylum-level classification of bacterial phylotypes in CD, UC and non-IBD control patients showing significant reduction in the proportion of *Firmicutes *sequences in UC samples relative to non-IBD controls (_*_^a^) and disruption in *Firmicutes *to *Bacteroidetes *ratio in IBD patients relative to non-IBD controls (_*_^b^).

There was a large degree of inter-individual variation between patients at all taxonomic levels but, despite this, distributions could be significantly associated with disease. Samples from both the inflamed and non-inflamed sites from CD and UC patients contained proportionally less *Firmicutes*, and correspondingly more *Bacteroidetes*, than the non-IBD control samples (Figure 2). The decreased proportion of *Firmicutes *present in UC, but not CD, samples reached statistical significance when compared with the controls (Figure 2). Related to these shifts, the ratio between *Firmicutes *and *Bacteroidetes *was changed in IBD patients. In non-IBD controls there were significantly more *Firmicutes *than *Bacteroidetes*, but this difference was lost with disease (Figure 2). We also observed a slight increase in *Enterobacteriaceae *in CD samples. *Enterobacteriaceae *were detected in 2 out of the 5 control patients and accounted for 0.11% of the total pooled community from these samples; they were detected in samples from 2 out of 6 UC patients and accounted for 0.09% of the total pooled community from these samples. In contrast, *Enterobacteriaceae *were detected in the paired biopsy samples from 5 out of the 6 CD patients included in the study and accounted for a ten-fold increase in proportion of the total CD microbiota compared to the other sample types (1.05%). This increase was significant when compared to UC samples (p = 0.049) but did not reach significance when compared to the non-IBD control cohort (p = 0.069). We could find no significant association, however, between microbiota composition and the severity of inflammation or the site of mucosal biopsy.

### Measurements of bacterial diversity

**Figure 3:**
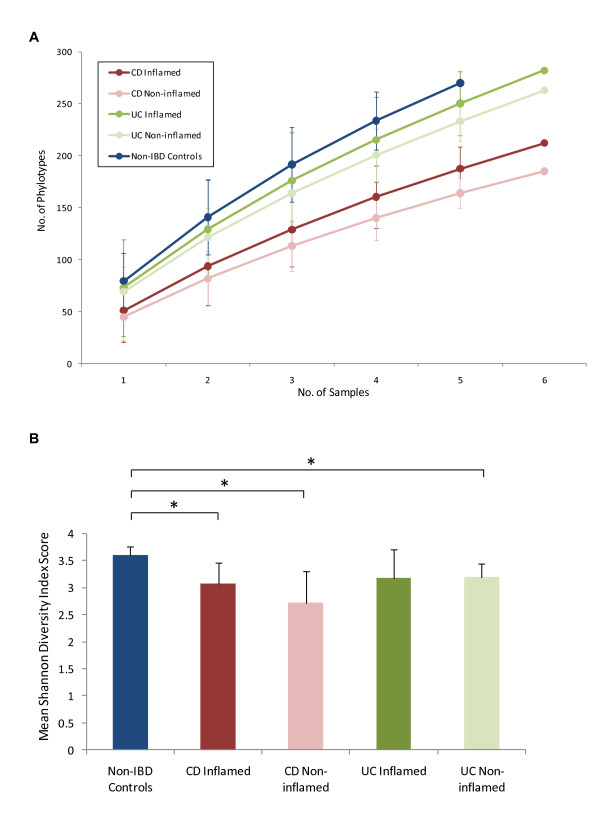
**Measures of bacterial diversity in the mucosal biopsies**. 3A) Rarefaction analysis showing number of phylotypes observed with increasing sequencing effort across all patient cohorts. Data points show the observed diversity after each individual biopsy sample was incorporated into the analysis. Colour-coded errors bars show 95% confidence intervals for each patient cohort. Note that, as each patient is incorporated into the analysis, the gap between the number of phylotypes observed in non-IBD patients compared to IBD patients grows larger. The reduction in species richness appeared to be particularly significant in CD patients. Number of sequences per sample: Non-IBD controls = 252-489, CD Inflamed = 248-342, CD Non-inflamed = 287-445, UC Inflamed = 267-469, UC Non-inflamed = 286-499. 3B) Mean Shannon diversity indices (SDI) calculated from the individual biopsies for each sample type. Significantly reduced SDI compared to non-IBD control samples are indicated by * (p = < 0.05). Error bars indicate standard deviation from the mean.

Using a number of different measures to explore the bacterial diversity within our samples we found that there was reduced diversity in biopsies from IBD patients compared to controls and that the reduction was particularly apparent in patients with CD (Figure 3). Rarefaction curves built from the cumulative dataset revealed that there were significant differences in species richness between control and CD samples (Figure 3A). The rarefaction curves also revealed a trend towards a slight increase in species richness in inflamed versus non-inflamed tissues, although these difference were not significant. In agreement with these findings, using the Shannon diversity index (SDI) to measure the richness and evenness of each sample, we found that the individual non-IBD control samples generally generated the highest SDI figures and that these were significantly higher (p < 0.05) than those from both the inflamed and non-inflamed CD samples and from the non-inflamed UC samples (Figure 3B).

### Bacterial community structure comparisons

**Figure 4:**
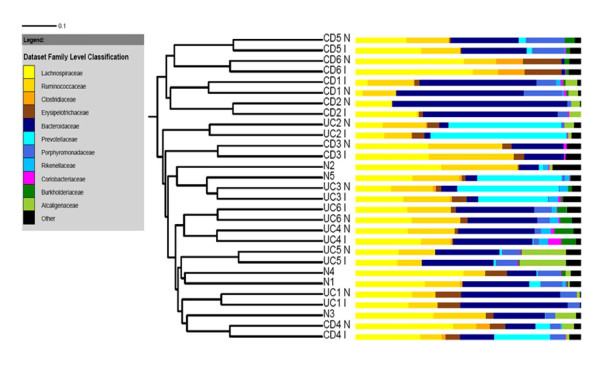
**Cluster dendrogram generated using the Jaccard coefficient, illustrating relationship between bacterial species membership and biopsy type across all samples included in the study**. Crohn's disease patients are indicated by numbers CD1-CD6. Ulcerative colitis patients are indicated by UC1-UC6. Samples marked with "I" are from inflamed intestinal regions, those marked with "N" are from non-inflamed regions. Non-IBD control samples are indicated with N1-N5. Adjacent bar charts show the Family level classification (as determined by the RDP classifier) for each of the sequences per sample. Families coloured in yellow/brown belong to the *Firmicutes *phylum, blue = *Bacteroidetes*, pink = *Actinobacteria*, green = *Proteobacteria*, black = all other sequences not belonging to the specified Families.

**Figure 5:**
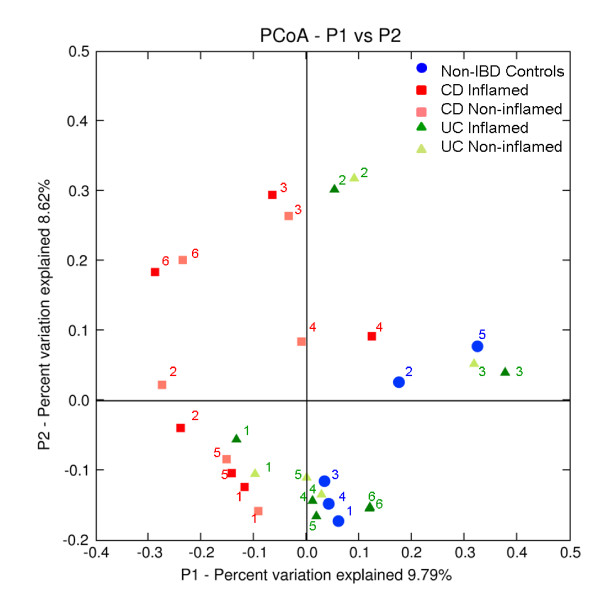
**Principal coordinates analysis of variation between the bacterial communities present in all biopsy samples**. Each data point represents an individual sample. Blue circles denote non-IBD control samples, red squares are Crohn's disease samples, green triangles are ulcerative colitis samples. Numbers indicate the donor the samples were obtained from. The paired, inflamed and non-inflamed, biopsy samples from each donor can be seen to cluster together. Figure was calculated using unweighted Fast UniFrac [39].

We next wanted to test whether or not the biopsy samples grouped together by disease cohort, by individual or both. Cluster analysis using both the Jaccard coefficient and PCoA showed that the samples clustered together according to donor (Figures 4 and 5) and that there was no separation between the CD, UC and non-IBD cohorts. There was also no separation based upon the location of biopsy sampling. This suggests that, despite differences in bacterial community composition and diversity between IBD and non-IBD samples, inter-individual variation is a stronger determinant of overall gut bacterial composition than disease. Despite this, although the paired samples clustered together, the branch lengths in the dendrogram were longer than might be expected if the community structure was highly similar between paired biopsies, indicating that there were still significant differences between the inflamed and non-inflamed tissues.

### Statistical comparisons between inflamed and non-inflamed tissue

We therefore sought to properly determine whether or not a characteristic localised dysbiosis between healthy and inflamed tissue within individual IBD patients exists. To test this we first performed whole community comparisons using ∫-LIBSHUFF [38], unweighted and weighted UniFrac [39] and the parsimony P-test [40] which all test whether or not two communities are significantly different overall without indicating which phylotypes cause the significance. We then used the Library Compare tool at the RDPII website [41], which pinpoints significant differences between two communities at all taxonomic designations from phylum to genus level to try and discover which bacterial groups were differentially abundant between the paired samples. Analyses with these tools indicated that in 11 out of the 12 IBD patients robust statistically significant differences between the inflamed and non-inflamed mucosal communities existed (Table 2).

**Table 2:** Comparison of bacterial composition from inflamed and non-inflamed tissue within individual IBD patients using ∫-LIBSHUFF, unweighted and weighted UniFrac, the parsimony P-test and RDP Library Compare.

	**Crohn's Disease Patients**						**Ulcerative Colitis Patients**					
	
	**CD1**	**CD2**	**CD3**	**CD4**	**CD5**	**CD6**	**UC1**	**UC2**	**UC3**	**UC4**	**UC5**	**UC6**
	
**∫-LIBSHUFF**	*	**	**	n/s	*	n/s	*	*	***	**	*	**
**UW UniFrac**	***	***	***	n/s	***	***	***	***	**	***	***	***
**W UniFrac**	**	***	n/s	***	*	n/s	n/s	***	***	**	n/s	*
**P-Test**	***	***	***	***	***	***	***	***	***	***	***	***
**Library Compare**												
***Firmicutes***	↑**	↑***		↓***					↑***			
>*Clostridiales*		↑***		↓***					↑***			
>>*Lachnospiraceae*		↑***		↓***								
>>*Ruminococcaceae*										↓**		
***Bacteroidetes***	↓**	↓***		↑***					↓***			
>>>*Bacteroides*		↓***										
>>>*Parabacteroides*	↓**											
>>>*Prevotella*				↑***				↑***	↓***			
***Actinobacteria***										↑**		
>>>*Collinsella*										↑**		
***Proteobacteria***				↓***								
>>>*Sutterella*				↓***								
***Fusobacteria***					↑*							
>>>*Fusobacterium*					↑*							

Key:- *** = P < 0.001, ** = P < 0.01, * = P < 0.05, n/s = P > 0.05. ↑ = Increased in inflamed vs. non-inflamed tissue, ↓ = Decreased in inflamed vs. non-inflamed tissue. **Bold **= Phylum level classification, > = Order level classification, >> = Family level classification, >>> = Genus level classification.

∫-LIBSHUFF analysis indicated a significant difference in all of the UC patients and 4 out 6 CD patients. Library Compare analysis confirmed that there were statistically significant differences between inflamed and non-inflamed sites for most of these samples. However, no obvious pattern was apparent and the statistically significant differences were spread between a number of phylogenetic groups (Table 2). Three of the sample pairs that had significant comparisons with ∫-LIBSHUFF (CD3, UC1 and UC5) showed no significant differences with Library Compare. Interestingly, these discrepancies may be explained by the UniFrac analysis. Unweighted UniFrac does not take into account the relative abundances of different phylotypes when comparing communities, only the species overlap. Weighted UniFrac also takes into account the relative abundance of each species. For the three sample pairs with no significant Library Compare results the unweighted UniFrac comparison showed highly significant differences between the paired communities, while the weighted comparison did not (Table 2). This indicates that these paired samples had significantly different community membership but that the overlapping members of the bacterial community that were present in both samples had similar abundances, thus explaining the significant ∫-LIBSHUFF results and the non-significant Library Compare results. In contrast to this, the paired set of samples from CD patient 4 were highly significantly different when measured using weighted UniFrac but showed no significance when measured using the unweighted version. Further analysis revealed that a *Prevotella *species was 3.6 times more abundant in the inflamed than non-inflamed site and accounted for 25% of the total community in the inflamed sample, a difference that was found to be significant to p < 0.00000001 with Library Compare. As the two communities were not recognised as significantly different with ∫-LIBSHUFF and unweighted UniFrac it is possible that this was because, regardless of the differential abundance, overall community membership was similar across both samples. The only sample pair to show no significant differences between inflamed and non-inflamed tissue with either ∫-LIBSHUFF or Library Compare (patient CD6) was characterised by a very low overall diversity, indicating that the microbiota may have been particularly disturbed in this patient.

As Library Compare is only able to classify sequences down to the genus level we then sought to characterise whether or not there were differences at the species level. For this purpose we compared sequences that had been grouped into phylotypes using DOTUR (99% identity) and assigned identities with MegaBLAST (see Additional file 1 ). While we were often able to observe statistically significant differences between individual phylotypes in single patients (data not shown) we were unable to detect a specific or recurring pattern or identify disease-specific phylotypes. Recently, a reduction in *Faecalibacterium prausnitzii *has been implicated in CD aetiology [31,42]. We did not observe a difference in *F. prausnitzii *proportional abundance between healthy and IBD patients but found that, when looking at paired biopsies from individual IBD patients, this species was almost always reduced in inflamed versus non-inflamed tissue. This trend did not reach statistical significance however. Species-level analysis also failed to identify any pathogenic species that have been previously associated with IBD such as *Mycobacterium avium *subspecies *paratuberculosis*, *Yersinia *spp or *Listeria *spp. [43]. We did recover *E. coli/Shigella *spp. from many CD samples but as 16S rRNA gene sequence data does not provide enough resolution to differentiate between commensal and pathogenic strains we could not determine whether or not these species were pathogenic. Sulphate-reducing bacteria (SRB) have also been implicated in the pathogenesis of IBD [44] but we recovered only one SRB sequence, which had greater than 99% identity to *Desulfovibrio piger*, and this was detected in one of the non-IBD control patients.

## Discussion

To our knowledge, this is one of the largest clone library studies investigating the microbiota in IBD. In contrast to an earlier study by Frank *et al*., [30], which examined a smaller number of clones from a large number of patients, we sought instead to add to current knowledge by obtaining a higher resolution of the IBD-associated microbiota with particular emphasis placed on observing differences between inflamed and non-inflamed colon sites in the same patients. This was inevitably done in a smaller number of patients and samples because of the depth of molecular analysis required for each sample. Our in-depth clone library analysis, utilizing the resolving power of near full-length 16S rRNA gene sequences, revealed significant differences in diversity and composition between the mucosal microbiota of healthy patients and IBD sufferers. The results also suggest a tendency towards a reduction in *Firmicutes *and an increase in *Bacteroidetes *species in IBD patients compared to controls and also indicate that there is an increase in *Enterobacteriaceae *in CD. Similar shifts in composition, in either one or all of these groups, have been reported by other investigators using both culture [22] and a variety of molecular techniques [29,31,45-55]. A previous large-scale clone library analysis by Frank *et al*., [30], however, reported a decrease in proportions of *Bacteroidetes *and the *Firmicutes *family *Lachnospiraceae *in a subset of, but not all, IBD patients and an increase in *Proteobacteria*. The observed discrepancies between these two large-scale clone library studies may in part be explained by different disease phenotypes, dietary or other environmental differences, the effect of inter-individual variation between patients or the differing number of samples studied and the depth of sequencing between each study.

We also demonstrated a reduction in bacterial diversity within IBD patients compared to controls and this is in agreement with several previous studies [24-27,56,57]. Our data shows, however, that despite the differences between IBD and non-IBD patients in both bacterial composition and diversity that samples clustered predominantly by individual rather than disease.

Using both culture and molecular methods, many studies have demonstrated that the mucosal community along the length of the colon is largely stable, in healthy and IBD patients, and distinct from that recovered in faeces [32-37]. Here we provide evidence instead for the development of localised differences in mucosal microbiota structure in IBD. Our community comparison results suggest that there may be differences between inflamed and non-inflamed tissue, with significant changes in the composition of the bacterial communities at these sites. A number of prior studies have also attempted to establish whether or not there is localised dysbiosis in IBD between inflamed and non-inflamed tissue. While two of these studies indicated that there is a dysbiosis [58,59], the majority have suggested that this is not the case [29,48,60-62]. Discrepancies between these results and ours may result from the use of differing molecular methodology and/or the greater sequencing depth we employed. DGGE/TGGE and FISH are useful tools but the resolving power of these methods is much lower than that for in-depth clone libraries covering the full length of the 16S rRNA gene [63]. In addition, DGGE/TGGE cannot accurately describe quantitative differences between dominant bands or describe qualitative differences in sub-dominant species and single bands on the gel may contain DNA from more than one species [64].

While our results suggest that localised changes in the mucosal microbiota do exist in IBD we were not able to identify a bacterial species or cluster that was consistently associated with the inflamed gut and therefore, potentially, with IBD aetiology. Other large-scale clone library analyses have also failed to identify specific pathogens [29,30]. While their absence may indicate that potential pathogens may simply form a very minor component of the microbiota, these results do not support the hypothesis that a particular bacterial agent causes IBD. Clone libraries generate inherent biases, however, and it is possible that they are unable to detect certain species due to methodological artefacts. Indeed, this may be important with *Mycobacterium avium *subspecies *paratuberculosis*, a member of the often underrepresented *Actinobacteria *phylum [65,66]. The absence of bifidobacteria from our dataset indicates that our clone libraries also suffer from this same bias against *Actinobacteria*. It is also worth noting that our analysis would not detect any viral, archaeal or eukaryotic aetiological agents. This may be important given recent evidence suggesting a role for viruses in the induction of at least some models of IBD [67].

Sequence-based microbiota comparisons such as ours can of course only demonstrate associations and do not provide information regarding mechanism or causation. It is also difficult to differentiate between compositional changes that may play a role in disease pathogenesis and those which may simply have occurred as a result of disease. However, given the absence of a specific and recurring aetiological agent in the cumulative data across all published IBD studies, which incorporate both culture- and molecular-based methodologies, it is possible that the alterations in bacterial composition and diversity seen between healthy and IBD patients and between inflamed and non-inflamed mucosa may be, to at least some extent, the result of the disturbed gut environment rather than the direct cause of disease. Indeed, there are a number of reasons why IBD is likely to result in altered conditions for bacterial growth. For example, the gut in IBD is likely to be a less stable environment than that of healthy individuals, with more exposure to antibiotics and other drug regimes, and alterations in transit time. Microscopy studies have suggested that there is a higher penetration of bacteria and a greater bacterial load in the mucosal layer in IBD patients [47,68] and the resulting inflammation drives the localised release of antimicrobial compounds [69]. In addition, in UC there is a reduced mucus layer in inflamed relative to non-inflamed regions [70].

Despite proportional increases in *Enterobacteriaceae *and *Bacteroidetes *within IBD patients, if these organisms were directly responsible for disease we might expect them to be elevated at sites of inflammation and this was not shown in our analysis. Taking into account all of the above factors, the observed increases in these bacterial groups in IBD patients as a whole may therefore simply reflect the adaptation of the individual microbiota to the IBD gut environment. *Bacteroides thetaiotaomicron*, for example, can adapt to inflammation in an experimental mouse model by inducing genes that metabolise host oxidative products [71] and inflammation *per se *has also been shown to promote the growth of *Enterobacteriaceae *in mouse models [72,73]. Clearly, further similar studies are required on a far greater range of gut bacterial species so that we can better understand the response of the gut microbiota to alterations in environmental conditions.

## Conclusions

This work demonstrates a dysbiosis, or imbalance, in microbial community structure and composition in inflammatory bowel disease patients relative to non-IBD controls. It also indicates that inflamed tissue differs from non-inflamed tissue, but not in a consistent or predictable manner. Indeed, despite general trends such as a reduction in diversity, the response to IBD may be to some extent specific to the individual. This lends support to the emerging hypothesis that IBD is combinatorial in aetiology, with many different combinations of genetic and environmental causes leading to similar therapeutic responses [67], and highlights the importance of interconnection between the environment, the microbiota and the host in health and disease.

Despite this, even if particular bacteria are not the specific cause of IBD, altered immune responses may act to select particular bacterial species through creation of favourable microenvironments and might therefore cause the outgrowth of potentially pathogenic commensal species [74]. Shifts in the microbiota may therefore still impact gut health by altering the antigenic exposure to the gut mucosa or by reducing its exposure to beneficial microbes and/or their metabolic products, thereby initiating a cycle that favours recruitment and growth of more pro-inflammatory species [17,75]. The observed reduction in *Firmicutes *proportions, for example, might lead to an undesirable affect on gut health. Recent work describing the anti-inflammatory properties of one *Firmicutes *species, *Faecalibacterium prausnitzii *[42] illustrates this point.

Finally, results from metagenomic studies indicate that, regardless of species composition, the collective genomes of each individual's microbiota appear to encode a remarkably conserved set of functions [28]. If similar, and potentially aggravating, factors are encoded by multiple species, it is possible that we will be better served in the future by looking at the complete gene complement of the microbial community as a whole, not just species composition. With this in mind, it is hoped that further analysis of the complex interplay between host and microbes will yield important insights into the pathogenesis of IBD.

## Methods

### Patients

Patients were selected from those undergoing routine colonoscopic assessment of IBD at Guy's and St. Thomas' Hospitals, London, UK. As controls, asymptomatic individuals undergoing colonoscopy for a family history of colorectal cancer or polyp surveillance were also invited to take part. Written informed consent was obtained from each patient and the study was granted ethical approval by the St. Thomas' Research Ethics Committee (Ref No. 06/Q0702/74). Patient information, including sex, age and the location of the colon that biopsies were taken from, is given in Table 1.

Colonoscopy was undertaken after prior preparation of the colon with two sachets of sodium picosulphate. No individuals received antibiotics in the preceding 2 months. For those with CD or UC, mucosal biopsies were taken from macroscopically inflamed and non-inflamed areas of the colon using standard gape forceps. Once taken, biopsy samples (approximately 1 × 2 mm) were placed in a cryovial without preservative, immediately snap frozen in liquid nitrogen, and stored at -70°C until analysis. Additional biopsy samples from the same area were also sent for histological analysis. These biopsies were scored independently for presence of ulceration, acute and chronic inflammation by a single gastrointestinal pathologist. Prior diagnosis of active CD or UC was determined by standard clinical, radiological, endoscopic and histopathological criteria. A modified Baron score with a range from 0-5, where a score of 5 represents the most severe disease, was used to grade the endoscopic severity of inflammation at the site of each biopsy used in the study [76].

### DNA extraction and sequence analysis

DNA was extracted from each mucosal biopsy sample using the QIAamp^® ^DNA Mini-Kit (Qiagen, UK) and the eluted DNA was stored at -20°C. 16S rRNA genes were amplified using the broad-range bacterial primers Bact-8F (5'-AGAGTTTGATCCTGGCTCAG-3') and Bact-1391R (5'-GACGGGCGGTGTGTRCA-3') [34]. Clone library construction and sequencing were carried out as described previously [72].

Sequences were aligned using the NAST aligner [77] and these alignments were subject to extensive manual curation using the ARB package [78] before further analysis. Sequences were tested for chimeras with Mallard [79], Bellerophon at Greengenes [77] and Pintail [80] and any that appeared to be chimeric were removed. The sequences (deposited in GenBank under accession numbers FJ503060-FJ513069) were initially given a broad classification to the phylum and family levels using the Classifier tool at the RDPII website [41]. To obtain more detailed taxonomic information the sequences were then divided into phylotypes. Distance matrices were generated in ARB with the Olsen correction and a 60% maximal-base frequency filter applied. This filter removed many ambiguously-aligned columns but was not so stringent that distinct species were commonly merged into single phylotypes. Distance matrices were then entered into the DOTUR program [81] set to the furthest neighbour and 99%-similarity setting. The resulting phylotypes were then assigned similarities to nearest neighbours using MegaBLAST [82].

To determine the depth of coverage in each of the clone libraries Good's coverage was calculated using the mothur software package [40]. Using this estimator the median coverage across all samples was found to be 94.35% (range of 83.73-97.3%).

Shannon diversity indices were calculated for each library by entering distance matrices generated in ARB, with the Olsen correction and a 60% maximal base-frequency filter applied, into DOTUR [81]. Rarefaction curves for each sample were calculated using mothur [40].

Community structure comparisons across the whole dataset, incorporating unweighted and weighted UniFrac, Parsimony testing and cluster analysis using the Jaccard coefficient, were performed using mothur and were based on an alignment created in mothur using the reference SILVA-alignment and with the 60% maximal-base filter and Olsen correction applied prior to distance matrix construction in ARB. Cluster dendrograms, with added bar charts showing the microbial composition of each sample, were visualised using the iTOL web package [83].

Paired (inflamed and non-inflamed) biopsy sample sequences from individual patients were aligned using the NAST aligner and were again extensively corrected in the ARB package [78] before further analysis. Olsen-corrected, 60% maximal-base frequency filtered distance matrices were subjected to ∫-LIBSHUFF analysis [38]. Unaligned paired-sample sequences were used as input for the Library Compare tool at the RDPII website [41].

Principal coordinates analysis (PCoA) plots were generated using the Fast UniFrac web application [39] based upon neighbour joining trees created in ARB, with 60% maximal-base frequency filter and Olsen correction applied, using the sequences aligned to the SILVA reference in mothur as initial input.

### Quantitative PCR (qPCR)

Total bacteria were quantified in 25 of the 29 biopsies by qPCR (CD1 non-inflamed, CD5 inflamed, CD5 non-inflamed and UC4 non-inflamed were not included in the analysis due to a lack of DNA from these samples). All PCRs were performed using a Stratagene Mx3000P thermal cycler, in conjunction with Stratagene MxPro qPCR Software. Each reaction contained a total volume of 20 μl per well and was performed in triplicate. qPCR reactions contained 10 ng of forward and reverse primer, 10 μl Brilliant II SYBR Green qPCR Master Mix (Agilent Technologies, La Jolla, CA), ~ 900 pg of template DNA (1:100 dilutions of sample genomic DNA preparations) and were made up to 20 μl with RNase free water. A 466-bp fragment of the bacterial 16S rRNA gene was amplified using the forward primer 5'-TCCTACGGGAGGCAGCAGT-3' and the reverse primer 5' -GGACTACCAGGGTATCTAATCCTGTT-3' [84]. The thermal cycling conditions were 50°C for 2 minutes and 95°C for 5 minutes followed by 40 cycles of denaturing at 95°C for 15 seconds, primer annealing at 60°C for 30 seconds and DNA extension at 72°C for 90 seconds. Finally a dissociation step was added to qualitatively assess reaction product specificity (temperature raised to 95°C, cooled to 60°C then slowly heated back to 95°C) for melt curve analysis of the PCR products. Extracted DNA from a pure *Bacteroides vulgatus *(ATCC 8482) culture was prepared into a series of ten-fold dilutions in RNase free water ranging from 1 × 10^6 ^copies to one copy and used as a positive control in order to make a standard curve. Quantification of template concentrations was made by linear extrapolation of baseline-subtracted data from the bacterial dilution series standard curve. For each reaction a threshold of luminescence was determined and compared to the standard curve. Thus for each sample an equivalent concentration given in colony forming units could be established.

### Statistical analysis

For the qPCR and compositional results the Mann-Whitney U test was used for comparisons between two groups and the Kruskall-Wallace method, analogous to one-way analysis of variance, to compare more than two groups. The levels of significance reported were not adjusted to take account of multiple comparisons. As these were multiple comparisons, p values <1% were considered significant to imply strong evidence of a difference.

## Authors' contributions

AWW carried out the clone library construction, performed the sequence analysis and drafted the manuscript. CC co-ordinated the sequencing. JDS, GCP and BH were involved in recruitment of patients and samples for the study. LP performed the qPCR analysis, carried out clone library construction and was involved in the sequence analysis. JDS, GCP, NR, BNH, JB, JP, GD and LP conceived of the study, participated in its design and coordination and helped to draft the manuscript. All authors read and approved the final manuscript.

## Supplementary Material

Additional File 1 **Species-level analysis of mucosa-associated microbiota at inflamed and non-inflamed sites within individual patients and within non-IBD controls**. Phylotypes generated using DOTUR (99% identity) were assigned identities with MegaBLAST. Phylotypes were given the name of the closest-matching environmental clone in the NCBI database and also the closest cultured relative. If closest matching identities were >99% these were not indicated in the figure, identities <99% are shown in brackets. The bacterial phyla individual phylotypes were mapped to are indicated by the coloured boxes. Click here for file

## References

[B1] Loftus EV Clinical epidemiology of inflammatory bowel disease: Incidence, prevalence, and environmental influences Gastroenterology 2004 126 1504 1517 10.1053/j.gastro.2004.01.063 15168363

[B2] Pizzi LT Weston CM Goldfarb NI Moretti D Cobb N Howell JB Infantolino A Dimarino AJ Cohen S Impact of chronic conditions on quality of life in patients with inflammatory bowel disease Inflamm Bowel Dis 2006 12 47 52 10.1097/01.MIB.0000191670.04605.e7 16374258

[B3] Halfvarson J Bodin L Tysk C Lindberg E Järnerot G Inflammatory bowel disease in a Swedish twin cohort: a long-term follow-up of concordance and clinical characteristics Gastroenterology 2003 124 1767 1773 10.1016/S0016-5085(03)00385-8 12806610

[B4] Barrett JC Hansoul S Nicolae DL Cho JH Duerr RH Rioux JD Brant SR Silverberg MS Taylor KD Barmada MM Bitton A Dassopoulos T Datta LW Green T Griffiths AM Kistner EO Murtha MT Regueiro MD Rotter JI Schumm LP Steinhart AH Targan SR Xavier RJ NIDDK IBD Genetics Consortium Libioulle C Sandor C Lathrop M Belaiche J Dewit O Gut I Genome-wide association defines more than 30 distinct susceptibility loci for Crohn's disease Nat Genet 2008 40 955 962 10.1038/ng.175 18587394 PMC2574810

[B5] Xavier RJ Podolsky DK Unravelling the pathogenesis of inflammatory bowel disease Nature 2007 448 427 434 10.1038/nature06005 17653185

[B6] Sartor RB Pathogenesis and immune mechanisms of chronic inflammatory bowel diseases Am J Gastroenterol 1997 92 12 Suppl 5S 11S 9395346

[B7] Bouma G Strober W The immunological and genetic basis of inflammatory bowel disease Nat Rev Immunol 2003 3 521 533 10.1038/nri1132 12876555

[B8] Cho JH The genetics and immunopathogenesis of inflammatory bowel disease Nat Rev Immunol 2008 8 458 466 10.1038/nri2340 18500230

[B9] Ley RE Lozupone CA Hamady M Knight R Gordon JI Worlds within worlds: evolution of the vertebrate gut microbiota Nat Rev Micro 2008 6 776 788 10.1038/nrmicro1978 PMC2664199 18794915

[B10] Dethlefsen L Eckburg PB Bik EM Relman DA Assembly of the human intestinal microbiota Trends Ecol Evol 2006 21 517 523 10.1016/j.tree.2006.06.013 16820245

[B11] Tlaskalová-Hogenová H Stepánková R Hudcovic T Tucková L Cukrowska B Lodinová-Zádníková R Kozáková H Rossmann P Bártová J Sokol D Funda DP Borovská D Reháková Z Sinkora J Hofman J Drastich P Kokesová A Commensal bacteria (normal microflora), mucosal immunity and chronic inflammatory and autoimmune diseases Immunol Lett 2004 93 97 108 15158604 10.1016/j.imlet.2004.02.005

[B12] Canny GO McCormick BA Bacteria in the intestine, helpful residents or enemies from within? Infect Immun 2008 76 3360 3373 10.1128/IAI.00187-08 18474643 PMC2493210

[B13] Harper PH Lee EC Kettlewell MG Bennett MK Jewell DP Role of the faecal stream in the maintenance of Crohn's colitis Gut 1985 26 279 284 10.1136/gut.26.3.279 3972275 PMC1432617

[B14] Nell S Suerbaum S Josenhans C The impact of the microbiota on the pathogenesis of IBD: lessons from mouse infection models Nat Rev Microbiol 2010 8 564 577 10.1038/nrmicro2403 20622892

[B15] Schultsz C Van Den Berg FM Ten Kate FW Tytgat GN Dankert J The intestinal mucus layer from patients with inflammatory bowel disease harbors high numbers of bacteria compared with controls Gastroenterology 1999 117 1089 1097 10.1016/S0016-5085(99)70393-8 10535871

[B16] Swidsinski A Ladhoff A Pernthaler A Swidsinski S Loening-Baucke V Ortner M Weber J Hoffmann U Schreiber S Dietel M Lochs H Mucosal flora in inflammatory bowel disease Gastroenterology 2002 122 44 54 10.1053/gast.2002.30294 11781279

[B17] Sartor RB Microbial influences in inflammatory bowel diseases Gastroenterology 2008 134 577 594 10.1053/j.gastro.2007.11.059 18242222

[B18] Rutgeerts P Hiele M Geboes K Peeters M Penninckx F Aerts R Kerremans R Controlled trial of Metronidazole treatment for prevention of Crohn's recurrence after ileal resection Gastroenterology 1995 108 1617 1621 10.1016/0016-5085(95)90121-3 7768364

[B19] Stringer EE Nicholson TJ Armstrong D Efficacy of topical Metronidazole (10 percent) in the treatment of anorectal Crohn's disease Dis Colon Rectum 2005 48 970 974 10.1007/s10350-004-0873-8 15785894

[B20] Feller M Huwiler K Stephan R Altpeter E Shang A Furrer H Pfyffer GE Jemmi T Baumgartner A Egger M *Mycobacterium avium *subspecies *paratuberculosis *and Crohn's disease: a systematic review and meta-analysis Lancet Infect Dis 2007 7 607 613 10.1016/S1473-3099(07)70211-6 17714674

[B21] Barnich N Darfeuille-Michaud A Adherent-invasive *Escherichia coli *and Crohn's disease Curr Opin Gastroenterol 2007 23 16 20 10.1097/MOG.0b013e3280105a38 17133079

[B22] Tamboli CP Neut C Desreumaux P Colombel JF Dysbiosis in inflammatory bowel disease Gut 2004 53 1 4 10.1136/gut.53.1.1 14684564 PMC1773911

[B23] Sartor RB Muehlbauer M Microbial host interactions in IBD: implications for pathogenesis and therapy Curr Gastroenterol Rep 2007 9 497 507 10.1007/s11894-007-0066-4 18377803

[B24] Ott SJ Musfeldt M Wenderoth DF Hampe J Brant O Fölsch UR Timmis KN Schreiber S Reduction in diversity of the colonic mucosa associated bacterial microflora in patients with active inflammatory bowel disease Gut 2004 53 685 693 10.1136/gut.2003.025403 15082587 PMC1774050

[B25] Manichanh C Rigottier-Gois L Bonnaud E Gloux K Pelletier E Frangeul L Nalin R Jarrin C Chardon P Marteau P Roca J Dore J Reduced diversity of faecal microbiota in Crohn's disease revealed by a metagenomic approach Gut 2006 55 205 211 10.1136/gut.2005.073817 16188921 PMC1856500

[B26] Scanlan PD Shanahan F O'Mahony C Marchesi JR Culture-independent analyses of temporal variation of the dominant fecal microbiota and targeted bacterial subgroups in Crohn's disease J Clin Microbiol 2006 44 3980 3988 10.1128/JCM.00312-06 16988018 PMC1698357

[B27] Martinez C Antolin M Santos J Torrejon A Casellas F Borruel N Guarner F Malagelada JR Unstable composition of the fecal microbiota in ulcerative colitis during clinical remission Am J Gastroenterol 2008 103 643 648 10.1111/j.1572-0241.2007.01592.x 18341488

[B28] Qin J Li R Raes J Arumugam M Burgdorf KS Manichanh C Nielsen T Pons N Levenez F Yamada T Mende DR Li J Xu J Li S Li D Cao J Wang B Liang H Zheng H Xie Y Tap J Lepage P Bertalan M Batto JM Hansen T Le Paslier D Linneberg A Nielsen HB Pelletier E Renault P A human gut microbial gene catalogue established by metagenomic sequencing Nature 2010 464 59 65 10.1038/nature08821 20203603 PMC3779803

[B29] Gophna U Sommerfeld K Gophna S Doolittle WF Veldhuyzen van Zanten SJ Differences between tissue-associated intestinal microfloras of patients with Crohn's disease and ulcerative colitis J Clin Microbiol 2006 44 4136 4141 10.1128/JCM.01004-06 16988016 PMC1698347

[B30] Frank DN St Amand AL Feldman RA Boedeker EC Harpaz N Pace NR Molecular-phylogenetic characterization of microbial community imbalances in human inflammatory bowel diseases Proc Natl Acad Sci USA 2007 104 13780 13785 10.1073/pnas.0706625104 17699621 PMC1959459

[B31] Sokol H Seksik P Furet JP Firmesse O Nion-Larmurier I Beaugerie L Cosnes J Corthier G Marteau P Doré J Low counts of *Faecalibacterium prausnitzii *in colitis microbiota Inflamm Bowel Dis 2009 15 1183 1189 10.1002/ibd.20903 19235886

[B32] Poxton IR Brown R Sawyerr A Ferguson A Mucosa-associated bacterial flora of the human colon J Med Microbiol 1997 46 85 91 10.1099/00222615-46-1-85 9003751

[B33] Zoetendal EG von Wright A Vilpponen-Salmela T Ben-Amor K Akkermans AD de Vos WM Mucosa-associated bacteria in the human gastrointestinal tract are uniformly distributed along the colon and differ from the community recovered from feces Appl Environ Microbiol 2002 68 3401 3407 10.1128/AEM.68.7.3401-3407.2002 12089021 PMC126800

[B34] Eckburg PB Bik EM Bernstein CN Purdom E Dethlefsen L Sargent M Gill SR Nelson KE Relman DA Diversity of the human intestinal microbial flora Science 2005 308 1635 1638 10.1126/science.1110591 15831718 PMC1395357

[B35] Wang M Ahrné S Jeppsson B Molin G Comparison of bacterial diversity along the human intestinal tract by direct cloning and sequencing of 16S rRNA genes FEMS Micro Ecol 2005 54 219 231 10.1016/j.femsec.2005.03.012 16332321

[B36] Lepage P Seksik P Sutren M de la Cochetière MF Jian R Marteau P Doré J Biodiversity of the mucosa-associated microbiota is stable along the distal digestive tract in healthy individuals and patients with IBD Inflamm Bowel Dis 2005 11 473 480 10.1097/01.MIB.0000159662.62651.06 15867587

[B37] Green GL Brostoff J Hudspith B Michael M Mylonaki M Rayment N Staines N Sanderson J Rampton DS Bruce KD Molecular characterization of the bacteria adherent to human colorectal mucosa J Appl Micro 2006 100 460 469 10.1111/j.1365-2672.2005.02783.x 16478485

[B38] Schloss PD Larget BR Handelsman J Integration of microbial ecology and statistics: a test to compare gene libraries Appl Environ Microbiol 2004 70 5485 5492 10.1128/AEM.70.9.5485-5492.2004 15345436 PMC520927

[B39] Hamady M Lozupone C Knight R Fast UniFrac: facilitating high-throughput phylogenetic analyses of microbial communities including analysis of pyrosequencing and PhyloChip data ISME J 2010 4 17 27 10.1038/ismej.2009.97 19710709 PMC2797552

[B40] Schloss PD Westcott SL Ryabin T Hall JR Hartmann M Hollister EB Lesniewski RA Oakley BB Parks DH Robinson CJ Sahl JW Stres B Thallinger GG Van Horn DJ Weber CF Introducing mothur: open-source, platform-independent, community-supported software for describing and comparing microbial communities Appl Environ Microbiol 2009 75 7537 7541 10.1128/AEM.01541-09 19801464 PMC2786419

[B41] Cole JR Wang Q Cardenas E Fish J Chai B Farris RJ Kulam-Syed-Mohideen AS McGarrell DM Marsh T Garrity GM Tiedje JM The Ribosomal Database Project: improved alignments and new tools for rRNA analysis Nucleic Acids Res 2009 37 Database D141 145 10.1093/nar/gkn879 19004872 PMC2686447

[B42] Sokol H Pigneur B Watterlot L Lakhdari O Bermúdez-Humarán LG Gratadoux JJ Blugeon S Bridonneau C Furet JP Corthier G Grangette C Vasquez N Pochart P Trugnan G Thomas G Blottière HM Doré J Marteau P Seksik P Langella P *Faecalibacterium prausnitzii *is an anti-inflammatory commensal bacterium identified by gut microbiota analysis of Crohn disease patients Proc Natl Acad Sci USA 2008 105 16731 16736 10.1073/pnas.0804812105 18936492 PMC2575488

[B43] Eckburg PB Relman DA The role of microbes in Crohn's disease Clin Infect Dis 2007 44 256 262 10.1086/510385 17173227

[B44] Loubinoux J Bronowicki J Pereira IAC Mougenel J Le Faou AE Sulfate-reducing bacteria in human feces and their association with inflammatory bowel diseases FEMS Micro Ecol 2002 40 107 112 10.1111/j.1574-6941.2002.tb00942.x 19709217

[B45] Seksik P Rigottier-Gois L Gramet G Sutren M Pochart P Marteau P Jian R Doré J Alterations of the dominant faecal bacterial groups in patients with Crohn's disease of the colon Gut 2003 52 237 242 10.1136/gut.52.2.237 12524406 PMC1774977

[B46] Mangin I Bonnet R Seksik P Rigottier-Gois L Sutren M Bouhnik Y Neut C Collins MD Colombel JF Marteau P Doré J Molecular inventory of faecal microflora in patients with Crohn's disease FEMS Micro Ecol 2004 50 25 36 10.1016/j.femsec.2004.05.005 19712374

[B47] Swidsinski A Weber J Loening-Baucke V Hale LP Lochs H Spatial organization and composition of the mucosal flora in patients with inflammatory bowel disease J Clin Microbiol 2005 43 3380 3389 10.1128/JCM.43.7.3380-3389.2005 16000463 PMC1169142

[B48] Bibiloni R Mangold M Madsen KL Fedorak RN Tannock GW The bacteriology of biopsies differs between newly diagnosed, untreated, Crohn's disease and ulcerative colitis patients J Med Microbiol 2006 55 1141 1149 10.1099/jmm.0.46498-0 16849736

[B49] Lucke K Miehlke S Jacobs E Schuppler M Prevalence of *Bacteroides *and *Prevotella *spp. in ulcerative colitis J Med Microbiol 2006 55 617 624 10.1099/jmm.0.46198-0 16585651

[B50] Martinez-Medina M Aldeguer X Gonzalez-Huix F Acero D Garcia-Gil LJ Abnormal microbiota composition in the ileocolonic mucosa of Crohn's disease patients as revealed by polymerase chain reaction-denaturing gradient gel electrophoresis Inflamm Bowel Dis 2006 12 1136 1145 10.1097/01.mib.0000235828.09305.0c 17119388

[B51] Sokol H Lepage P Seksik P Doré J Marteau P Temperature gradient gel electrophoresis of fecal 16S rRNA reveals active *Escherichia coli *in the microbiota of patients with ulcerative colitis J Clin Microbiol 2006 44 3172 3177 10.1128/JCM.02600-05 16954244 PMC1594675

[B52] Baumgart M Dogan B Rishniw M Weitzman G Bosworth B Yantiss R Orsi RH Wiedmann M McDonough P Kim SG Berg D Schukken Y Scherl E Simpson KW Culture independent analysis of ileal mucosa reveals a selective increase in invasive *Escherichia coli *of novel phylogeny relative to depletion of Clostridiales in Crohn's disease involving the ileum ISME J 2007 1 403 418 10.1038/ismej.2007.52 18043660

[B53] Kotlowski R Bernstein CN Sepehri S Krause DO High prevalence of Escherichia coli belonging to the B2+D phylogenetic group in inflammatory bowel disease Gut 2007 56 669 675 10.1136/gut.2006.099796 17028128 PMC1942160

[B54] Andoh A Tsujikawa T Sasaki M Mitsuyama K Suzuki Y Matsui T Matsumoto T Benno Y Fujiyama Y Fecal microbiota profile of Crohn's disease determined by terminal restriction fragment length polymorphism analysis Aliment Pharmacol Ther 2009 29 75 82 10.1111/j.1365-2036.2008.03860.x 18945264

[B55] Martinez-Medina M Aldeguer X Lopez-Siles M González-Huix F López-Oliu C Dahbi G Blanco JE Blanco J Garcia-Gil LJ Darfeuille-Michaud A Molecular diversity of *Escherichia coli *in the human gut: New ecological evidence supporting the role of adherent-invasive *E. coli *(AIEC) in Crohn's disease Inflamm Bowel Dis 2009 15 872 882 10.1002/ibd.20860 19235912

[B56] Dicksved J Halfvarson J Rosenquist M Järnerot G Tysk C Apajalahti J Engstrand L Jansson JK Molecular analysis of the gut microbiota of identical twins with Crohn's disease ISME J 2008 2 716 727 10.1038/ismej.2008.37 18401439

[B57] Ott SJ Plamondon S Hart A Begun A Rehman A Kamm MA Schreiber S Dynamics of the mucosa-associated flora in ulcerative colitis patients during remission and clinical relapse J Clin Microbiol 2008 46 3510 3513 10.1128/JCM.01512-08 18701655 PMC2566070

[B58] Mylonaki M Rayment NB Rampton DS Hudspith BN Brostoff J Molecular characterization of rectal mucosa-associated bacterial flora in inflammatory bowel disease Inflamm Bowel Dis 2005 11 481 487 10.1097/01.MIB.0000159663.62651.4f 15867588

[B59] Sepehri S Kotlowski R Bernstein CN Krause DO Microbial diversity of inflamed and noninflamed gut biopsy tissues in inflammatory bowel disease Inflamm Bowel Dis 2007 13 675 683 10.1002/ibd.20101 17262808

[B60] Seksik P Lepage P de la Cochetière MF Bourreille A Sutren M Galmiche JP Doré J Marteau P Search for localized dysbiosis in Crohn's disease ulcerations by temporal temperature gradient gel electrophoresis of 16S rRNA J Clin Microbiol 2005 43 4654 4658 10.1128/JCM.43.9.4654-4658.2005 16145122 PMC1234104

[B61] Sokol H Lepage P Seksik P Doré J Marteau P Molecular comparison of dominant microbiota associated with injured versus healthy mucosa in ulcerative colitis Gut 2007 56 152 154 10.1136/gut.2006.109686 17172591 PMC1856643

[B62] Vasquez N Mangin I Lepage P Seksik P Duong JP Blum S Schiffrin E Suau A Allez M Vernier G Tréton X Doré J Marteau P Pochart P Patchy distribution of mucosal lesions in ileal Crohn's disease is not linked to differences in the dominant mucosa-associated bacteria: a study using fluorescence in situ hybridization and temporal temperature gradient gel electrophoresis Inflamm Bowel Dis 2007 13 684 692 10.1002/ibd.20084 17206669

[B63] Bent SJ Forney LJ The tragedy of the uncommon: understanding limitations in the analysis of microbial diversity ISME J 2008 2 689 695 10.1038/ismej.2008.44 18463690

[B64] Marzorati M Wittebolle L Boon N Daffonchio D Verstraete W How to get more out of molecular fingerprints: practical tools for microbial ecology Environ Microbiol 2008 10 1571 1581 10.1111/j.1462-2920.2008.01572.x 18331337

[B65] Farris MH Olson JB Detection of Actinobacteria cultivated from environmental samples reveals bias in universal primers Lett Appl Microbiol 2007 45 376 381 10.1111/j.1472-765X.2007.02198.x 17897379

[B66] Frank JA Reich CI Sharma S Weisbaum JS Wilson BA Olsen GJ Critical evaluation of two primers commonly used for amplification of bacterial 16S rRNA genes Appl Environ Microbiol 2008 74 2461 2470 10.1128/AEM.02272-07 18296538 PMC2293150

[B67] Cadwell K Patel KK Maloney NS Liu TC Ng AC Storer CE Head RD Xavier R Stappenbeck TS Virgin HW Virus-plus-susceptibility gene interaction determines Crohn's disease gene *Atg16L1 *phenotypes in intestine Cell 2010 141 1135 1145 10.1016/j.cell.2010.05.009 20602997 PMC2908380

[B68] Kleessen B Kroesen AJ Buhr HJ Blaut M Mucosal and invading bacteria in patients with inflammatory bowel disease compared with controls Scand J Gastroenterol 2002 37 1034 1041 10.1080/003655202320378220 12374228

[B69] Winter SE Keestra AM Tsolis RM Bäumler AJ The blessings and curses of intestinal inflammation Cell Host Microbe 2010 8 36 43 10.1016/j.chom.2010.06.003 20638640 PMC2918243

[B70] Swidsinski A Loening-Baucke V Theissig F Engelhardt H Bengmark S Koch S Lochs H Dörffel Y Comparative study of the intestinal mucus barrier in normal and inflamed colon Gut 2007 56 343 350 10.1136/gut.2006.098160 16908512 PMC1856798

[B71] Peterson DA McNulty NP Guruge JL Gordon JI IgA response to symbiotic bacteria as a mediator of gut homeostasis Cell Host Microbe 2007 2 328 339 10.1016/j.chom.2007.09.013 18005754

[B72] Stecher B Robbiani R Walker AW Westendorf AM Barthel M Kremer M Chaffron S Macpherson AJ Buer J Parkhill J Dougan G von Mering C Hardt WD *Salmonella enterica *serovar Typhimurium exploits inflammation to compete with the intestinal microbiota PLoS Biol 2007 5 2177 2189 10.1371/journal.pbio.0050244 17760501 PMC1951780

[B73] Lupp C Robertson ML Wickham ME Sekirov I Champion OL Gaynor EC Finlay BB Host-mediated inflammation disrupts the intestinal microbiota and promotes the overgrowth of *Enterobacteriaceae* Cell Host Microbe 2007 2 119 129 10.1016/j.chom.2007.06.010 18005726

[B74] Artis D Epithelial-cell recognition of commensal bacteria and maintenance of immune homeostasis in the gut Nat Rev Immunol 2008 8 411 420 10.1038/nri2316 18469830

[B75] Kaser A Zeissig S Blumberg RS Inflammatory bowel disease Annu Rev Immunol 2010 28 573 621 10.1146/annurev-immunol-030409-101225 20192811 PMC4620040

[B76] Baron JH Connell AM Lennard-Jones JE Variation between observers in describing mucosal appearances in proctocolitis BMJ 1964 5375 89 92 10.1136/bmj.1.5375.89 PMC1812908 14075156

[B77] DeSantis TZ Hugenholtz P Larsen N Rojas M Brodie EL Keller K Huber T Dalevi D Hu P Andersen GL Greengenes, a chimera-checked 16S rRNA gene database and workbench compatible with ARB Appl Environ Microbiol 2006 72 5069 5072 10.1128/AEM.03006-05 16820507 PMC1489311

[B78] Ludwig W Strunk O Westram R Richter L Meier H Yadhukumar Buchner A Lai T Steppi S Jobb G Förster W Brettske I Gerber S Ginhart AW Gross O Grumann S Hermann S Jost R König A Liss T Lüssmann R May M Nonhoff B Reichel B Strehlow R Stamatakis A Stuckmann N Vilbig A Lenke M Ludwig T ARB: a software environment for sequence data Nucleic Acids Res 2004 32 1363 1371 10.1093/nar/gkh293 14985472 PMC390282

[B79] Ashelford KE Chuzhanova NA Fry JC Jones AJ Weightman AJ New screening software shows that most recent large 16S rRNA gene clone libraries contain chimeras Appl Environ Microbiol 2006 72 5734 5741 10.1128/AEM.00556-06 16957188 PMC1563593

[B80] Ashelford KE Chuzhanova NA Fry JC Jones AJ Weightman AJ At least 1 in 20 sequence records currently held in public repositories is estimated to contain substantial anomalies Appl Environ Microbiol 2005 71 7724 7736 10.1128/AEM.71.12.7724-7736.2005 16332745 PMC1317345

[B81] Schloss PD Handelsman J Introducing DOTUR, a computer program for defining operational taxonomic units and estimating species richness Appl Environ Microbiol 2005 71 1501 1506 10.1128/AEM.71.3.1501-1506.2005 15746353 PMC1065144

[B82] Johnson M Zaretskaya I Raytselis Y Merezhuk Y McGinnis S Madden TL NCBI BLAST: a better web interface Nucleic Acids Res 2008 36 Web server W5 W9 10.1093/nar/gkn201 18440982 PMC2447716

[B83] Letunic I Bork P Interactive Tree Of Life (iTOL): an online tool for phylogenetic tree display and annotation Bioinformatics 2007 23 127 128 10.1093/bioinformatics/btl529 17050570

[B84] Nadkarni MA Martin FE Jacques NA Hunter N Determination of bacterial load by real-time PCR using a broad-range (universal) probe and primers set Microbiology 2002 148 257 266 11782518 10.1099/00221287-148-1-257

